# Phenyl 3-meth­oxy-4-phen­oxy­benzoate

**DOI:** 10.1107/S1600536811033678

**Published:** 2011-08-27

**Authors:** Jing Zhou, Shuai Zheng, Gang Chen, Wenjing Wang, Zhenting Du

**Affiliations:** a College of Science, Northwest A&F University, Yangling, Shaanxi 712100, People’s Republic of China

## Abstract

In the title mol­ecule, C_20_H_16_O_4_, the two outermost phenyl rings form dihedral angles of 79.80 (7) and 69.35 (7)° with the central benzene ring. In the crystal structure, weak inter­molecular C—H⋯O inter­actions link the mol­ecules into ribbons propagating along [1
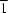
0].

## Related literature

For the general synthesis of derivatives of diphenyl­ethers, see: Paul & Gupta (2004 ▶). For related structures, see: Chen *et al.* (2006 ▶); Petek *et al.* (2005 ▶); Chantrapromma *et al.*(2001 ▶); Nakamura *et al.* (1983 ▶); Gopal *et al.* (1980 ▶). For applications of diphenyl­ether derivatives, see: Dey & Desiraju (2005 ▶); Wang *et al.* (2005 ▶). 
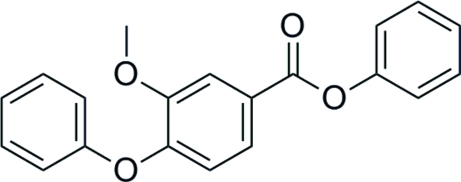


## Experimental

### 

#### Crystal data

C_20_H_16_O_4_
*M* *_r_* = 320.33
Monoclinic, 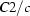

*a* = 11.0261 (10) Å
*b* = 11.9624 (11) Å
*c* = 24.961 (2) Å
β = 97.842 (1)°
*V* = 3261.5 (5) Å^3^
*Z* = 8
Mo *K*α radiation
μ = 0.09 mm^−1^
*T* = 298 K
0.49 × 0.42 × 0.40 mm

#### Data collection

Bruker SMART APEXII CCD area-detector diffractometer
Absorption correction: multi-scan (*SADABS*; Bruker, 2009 ▶) *T*_min_ = 0.957, *T*_max_ = 0.965
7972 measured reflections
2878 independent reflections
1785 reflections with *I* > 2σ(*I*)
*R*_int_ = 0.040

#### Refinement

*R*[*F* ^2^ > 2σ(*F* ^2^)] = 0.041
*wR*(*F* ^2^) = 0.118
*S* = 1.05
2878 reflections
219 parameters
H-atom parameters constrained
Δρ_max_ = 0.16 e Å^−3^
Δρ_min_ = −0.17 e Å^−3^

### 

Data collection: *APEX2* (Bruker, 2009 ▶); cell refinement: *SAINT* (Bruker, 2009 ▶); data reduction: *SAINT*; program(s) used to solve structure: *SHELXS97* (Sheldrick, 2008 ▶); program(s) used to refine structure: *SHELXL97* (Sheldrick, 2008 ▶); molecular graphics: *SHELXTL* (Sheldrick, 2008 ▶); software used to prepare material for publication: *SHELXTL*.

## Supplementary Material

Crystal structure: contains datablock(s) I, global. DOI: 10.1107/S1600536811033678/cv5133sup1.cif

Structure factors: contains datablock(s) I. DOI: 10.1107/S1600536811033678/cv5133Isup2.hkl

Supplementary material file. DOI: 10.1107/S1600536811033678/cv5133Isup3.cml

Additional supplementary materials:  crystallographic information; 3D view; checkCIF report

## Figures and Tables

**Table 1:** Hydrogen-bond geometry (Å, °)

*D*—H⋯*A*	*D*—H	H⋯*A*	*D*⋯*A*	*D*—H⋯*A*
C8—H8*C*⋯O2^i^	0.96	2.52	3.408 (4)	153
C14—H14⋯O2^ii^	0.93	2.53	3.446 (3)	167
C20—H20⋯O3^ii^	0.93	2.60	3.468 (3)	155

Symmetry codes: (i) 
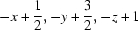
; (ii) 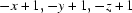.

**Fig. 1.**
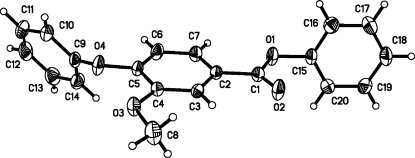
The molecular structure of (I). Displacement ellipsoids are drawn at the 30% probability level.

## supplementary crystallographic information

### Comment

4,4'-Dicarboxydiphenyl ether was widely used in polymer frameworks which show potential applications in microelectronics, nonlinear optics, porous materials and catalysis (Dey & Desiraju, 2005; Wang *et al.*, 2005). The title compound (I) was obtained as unexpected product in the cyclization of diazonium salt to dibenzofuran. Accidently, in our pursuing new methodology of synthesis of dibenzofuran the diazonium salt was heated without any catalyst. In order to determine the structure of a new compound, it was characterized by single-crystal X-ray analysis.

In (I) (Fig. 1) all bond lengths and angles are normal and correspond to those observed in the related compounds (Chen *et al.*, 2006; Petek *et al.*, 2005; Chantrapromma *et al.*, 2001; Nakamura *et al.*, 1983; Gopal *et al.*, 1980). Two utmost phenyl rings form the dihedral angles of 79.80 (7) and 69.35 (7)°, respectively, with the central benzene ring. In the crystal structure, weak intermolecular C—H···O interactions (Table 1) link molecules into ribbons propagated in [110].

### Experimental

A solution of 2-(2-methoxy-4-(phenoxycarbonyl)phenoxy)benzenediazonium tetrafluroborate (0.868 g, 2 mmol) in ethanol (30 ml) was refluxed for 1 h. Then the solvent was evaporated and the residue was purified through column chromatography. The compound was dissolved in hexane, and white square crystals were obtained by slow evaporation of the solvent over one week.

### Crystal data

**Table:**

C_20_H_16_O_4_	*D*_x_ = 1.305 Mg m^−^^3^
*M**_r_* = 320.33	Melting point: 383 K
Monoclinic, *C*2/*c*	Mo *K*α radiation, λ = 0.71073 Å
*a* = 11.0261 (10) Å	Cell parameters from 2372 reflections
*b* = 11.9624 (11) Å	θ = 2.5–27.3°
*c* = 24.961 (2) Å	µ = 0.09 mm^−^^1^
β = 97.842 (1)°	*T* = 298 K
*V* = 3261.5 (5) Å^3^	Block, white
*Z* = 8	0.49 × 0.42 × 0.40 mm
*F*(000) = 1344	

### Data collection

**Table:**

Bruker SMART APEXII CCD area-detector diffractometer	2878 independent reflections
Radiation source: fine-focus sealed tube	1785 reflections with *I* > 2σ(*I*)
graphite	*R*_int_ = 0.040
φ and ω scans	θ_max_ = 25.0°, θ_min_ = 2.5°
Absorption correction: multi-scan (*SADABS*; Bruker, 2009)	*h* = −12→13
*T*_min_ = 0.957, *T*_max_ = 0.965	*k* = −14→8
7972 measured reflections	*l* = −29→29

### Refinement

**Table:**

Refinement on *F*^2^	Secondary atom site location: difference Fourier map
Least-squares matrix: full	Hydrogen site location: inferred from neighbouring sites
*R*[*F*^2^ > 2σ(*F*^2^)] = 0.041	H-atom parameters constrained
*wR*(*F*^2^) = 0.118	*w* = 1/[σ^2^(*F*_o_^2^) + (0.0413*P*)^2^ + 1.7982*P*] where *P* = (*F*_o_^2^ + 2*F*_c_^2^)/3
*S* = 1.05	(Δ/σ)_max_ < 0.001
2878 reflections	Δρ_max_ = 0.16 e Å^−^^3^
219 parameters	Δρ_min_ = −0.17 e Å^−^^3^
0 restraints	Extinction correction: *SHELXL97* (Sheldrick, 2008), Fc^*^=kFc[1+0.001xFc^2^λ^3^/sin(2θ)]^-1/4^
Primary atom site location: structure-invariant direct methods	Extinction coefficient: 0.0053 (4)

### Special details

**Table:**

Geometry. All e.s.d.'s (except the e.s.d. in the dihedral angle between two l.s. planes) are estimated using the full covariance matrix. The cell e.s.d.'s are taken into account individually in the estimation of e.s.d.'s in distances, angles and torsion angles; correlations between e.s.d.'s in cell parameters are only used when they are defined by crystal symmetry. An approximate (isotropic) treatment of cell e.s.d.'s is used for estimating e.s.d.'s involving l.s. planes.
Refinement. Refinement of *F*^2^ against ALL reflections. The weighted *R*-factor *wR* and goodness of fit *S* are based on *F*^2^, conventional *R*-factors *R* are based on *F*, with *F* set to zero for negative *F*^2^. The threshold expression of *F*^2^ > σ(*F*^2^) is used only for calculating *R*-factors(gt) *etc*. and is not relevant to the choice of reflections for refinement. *R*-factors based on *F*^2^ are statistically about twice as large as those based on *F*, and *R*-factors based on ALL data will be even larger.

### Fractional atomic coordinates and isotropic or equivalent isotropic displacement parameters (Å^2^)

**Table:**

	*x*	*y*	*z*	*U*_iso_*/*U*_eq_	
O1	0.20235 (14)	0.40188 (12)	0.47155 (6)	0.0542 (5)	
O2	0.22859 (15)	0.58572 (13)	0.46169 (6)	0.0617 (5)	
O3	0.55526 (13)	0.68666 (12)	0.61835 (6)	0.0510 (4)	
O4	0.59538 (14)	0.50787 (12)	0.67467 (5)	0.0562 (5)	
C1	0.25750 (19)	0.50110 (18)	0.48580 (8)	0.0416 (5)	
C2	0.35069 (17)	0.49359 (16)	0.53388 (7)	0.0365 (5)	
C3	0.40974 (17)	0.59252 (17)	0.55134 (7)	0.0383 (5)	
H3	0.3923	0.6579	0.5316	0.046*	
C4	0.49363 (17)	0.59433 (16)	0.59748 (8)	0.0362 (5)	
C5	0.51873 (18)	0.49544 (17)	0.62679 (7)	0.0393 (5)	
C6	0.4624 (2)	0.39727 (18)	0.60920 (8)	0.0484 (6)	
H6	0.4809	0.3316	0.6285	0.058*	
C7	0.37782 (19)	0.39605 (18)	0.56250 (8)	0.0454 (6)	
H7	0.3396	0.3296	0.5506	0.054*	
C8	0.5401 (3)	0.7870 (2)	0.58760 (11)	0.0768 (9)	
H8A	0.5629	0.7742	0.5524	0.115*	
H8B	0.5911	0.8444	0.6056	0.115*	
H8C	0.4560	0.8101	0.5841	0.115*	
C9	0.6653 (2)	0.41886 (18)	0.69837 (8)	0.0459 (6)	
C10	0.6668 (2)	0.4066 (2)	0.75311 (9)	0.0601 (7)	
H10	0.6174	0.4510	0.7718	0.072*	
C11	0.7429 (3)	0.3271 (3)	0.77982 (10)	0.0760 (9)	
H11	0.7440	0.3173	0.8169	0.091*	
C12	0.8166 (2)	0.2624 (2)	0.75287 (12)	0.0739 (9)	
H12	0.8679	0.2094	0.7714	0.089*	
C13	0.8145 (2)	0.2764 (2)	0.69818 (11)	0.0663 (7)	
H13	0.8648	0.2329	0.6796	0.080*	
C14	0.7378 (2)	0.3548 (2)	0.67055 (9)	0.0566 (7)	
H14	0.7357	0.3638	0.6334	0.068*	
C15	0.1108 (2)	0.40450 (17)	0.42591 (8)	0.0436 (6)	
C16	−0.0096 (2)	0.40588 (19)	0.43430 (9)	0.0548 (6)	
H16	−0.0307	0.4064	0.4691	0.066*	
C17	−0.0987 (2)	0.4065 (2)	0.38964 (10)	0.0623 (7)	
H17	−0.1809	0.4077	0.3944	0.075*	
C18	−0.0671 (2)	0.4055 (2)	0.33860 (10)	0.0606 (7)	
H18	−0.1276	0.4068	0.3088	0.073*	
C19	0.0534 (2)	0.4024 (2)	0.33141 (9)	0.0620 (7)	
H19	0.0747	0.4013	0.2966	0.074*	
C20	0.1434 (2)	0.40105 (19)	0.37542 (9)	0.0539 (6)	
H20	0.2255	0.3978	0.3706	0.065*	

### Atomic displacement parameters (Å^2^)

**Table:**

	*U*^11^	*U*^22^	*U*^33^	*U*^12^	*U*^13^	*U*^23^
O1	0.0618 (10)	0.0448 (9)	0.0481 (9)	−0.0063 (8)	−0.0211 (8)	0.0062 (7)
O2	0.0733 (11)	0.0466 (10)	0.0555 (10)	−0.0032 (8)	−0.0256 (8)	0.0097 (8)
O3	0.0550 (9)	0.0386 (9)	0.0528 (9)	−0.0041 (7)	−0.0163 (7)	0.0042 (7)
O4	0.0706 (11)	0.0496 (10)	0.0405 (8)	0.0025 (8)	−0.0208 (8)	0.0034 (7)
C1	0.0434 (12)	0.0420 (13)	0.0374 (12)	−0.0009 (11)	−0.0016 (10)	0.0005 (10)
C2	0.0368 (11)	0.0383 (12)	0.0328 (10)	0.0004 (9)	−0.0008 (9)	0.0020 (9)
C3	0.0383 (11)	0.0392 (12)	0.0361 (11)	0.0030 (10)	0.0000 (9)	0.0093 (9)
C4	0.0348 (11)	0.0366 (12)	0.0359 (11)	0.0007 (9)	−0.0001 (9)	−0.0001 (9)
C5	0.0412 (12)	0.0430 (13)	0.0309 (10)	0.0019 (10)	−0.0053 (9)	0.0030 (9)
C6	0.0592 (14)	0.0394 (13)	0.0423 (12)	0.0007 (11)	−0.0086 (11)	0.0114 (10)
C7	0.0496 (13)	0.0397 (13)	0.0434 (12)	−0.0039 (10)	−0.0061 (10)	0.0018 (10)
C8	0.0853 (19)	0.0437 (15)	0.089 (2)	−0.0156 (14)	−0.0321 (16)	0.0169 (14)
C9	0.0454 (13)	0.0466 (13)	0.0403 (12)	−0.0045 (11)	−0.0131 (10)	0.0126 (11)
C10	0.0530 (14)	0.0817 (18)	0.0419 (13)	−0.0012 (13)	−0.0065 (11)	0.0126 (13)
C11	0.0707 (19)	0.104 (2)	0.0473 (15)	−0.0006 (18)	−0.0142 (14)	0.0313 (16)
C12	0.0597 (17)	0.078 (2)	0.0744 (19)	0.0020 (15)	−0.0244 (15)	0.0286 (16)
C13	0.0544 (15)	0.0668 (17)	0.0740 (18)	0.0055 (13)	−0.0047 (13)	0.0088 (14)
C14	0.0622 (16)	0.0592 (15)	0.0452 (13)	−0.0011 (13)	−0.0039 (12)	0.0090 (12)
C15	0.0481 (14)	0.0376 (12)	0.0405 (12)	−0.0027 (10)	−0.0108 (10)	0.0020 (10)
C16	0.0587 (16)	0.0583 (15)	0.0466 (13)	−0.0017 (12)	0.0044 (11)	−0.0078 (11)
C17	0.0422 (13)	0.0724 (18)	0.0694 (17)	0.0012 (12)	−0.0025 (12)	−0.0132 (14)
C18	0.0569 (16)	0.0643 (17)	0.0534 (15)	−0.0035 (13)	−0.0183 (12)	−0.0025 (12)
C19	0.0620 (17)	0.0809 (19)	0.0408 (13)	−0.0123 (14)	−0.0015 (11)	0.0036 (12)
C20	0.0456 (13)	0.0633 (16)	0.0514 (14)	−0.0077 (12)	0.0010 (11)	0.0023 (12)

### Geometric parameters (Å, °)

**Table:**

O1—C1	1.359 (2)	C9—C10	1.372 (3)
O1—C15	1.415 (2)	C10—C11	1.379 (3)
O2—C1	1.198 (2)	C10—H10	0.9300
O3—C4	1.362 (2)	C11—C12	1.364 (4)
O3—C8	1.422 (3)	C11—H11	0.9300
O4—C5	1.375 (2)	C12—C13	1.372 (4)
O4—C9	1.398 (2)	C12—H12	0.9300
C1—C2	1.472 (3)	C13—C14	1.383 (3)
C2—C7	1.379 (3)	C13—H13	0.9300
C2—C3	1.392 (3)	C14—H14	0.9300
C3—C4	1.375 (3)	C15—C20	1.358 (3)
C3—H3	0.9300	C15—C16	1.372 (3)
C4—C5	1.399 (3)	C16—C17	1.382 (3)
C5—C6	1.372 (3)	C16—H16	0.9300
C6—C7	1.390 (3)	C17—C18	1.366 (3)
C6—H6	0.9300	C17—H17	0.9300
C7—H7	0.9300	C18—C19	1.365 (3)
C8—H8A	0.9600	C18—H18	0.9300
C8—H8B	0.9600	C19—C20	1.377 (3)
C8—H8C	0.9600	C19—H19	0.9300
C9—C14	1.363 (3)	C20—H20	0.9300
			
C1—O1—C15	115.84 (15)	C9—C10—C11	118.6 (3)
C4—O3—C8	117.53 (15)	C9—C10—H10	120.7
C5—O4—C9	121.60 (16)	C11—C10—H10	120.7
O2—C1—O1	121.84 (18)	C12—C11—C10	121.1 (2)
O2—C1—C2	124.68 (19)	C12—C11—H11	119.4
O1—C1—C2	113.44 (18)	C10—C11—H11	119.4
C7—C2—C3	119.86 (17)	C11—C12—C13	119.4 (2)
C7—C2—C1	123.48 (19)	C11—C12—H12	120.3
C3—C2—C1	116.62 (17)	C13—C12—H12	120.3
C4—C3—C2	120.53 (18)	C12—C13—C14	120.4 (3)
C4—C3—H3	119.7	C12—C13—H13	119.8
C2—C3—H3	119.7	C14—C13—H13	119.8
O3—C4—C3	125.18 (17)	C9—C14—C13	119.2 (2)
O3—C4—C5	115.66 (16)	C9—C14—H14	120.4
C3—C4—C5	119.15 (18)	C13—C14—H14	120.4
C6—C5—O4	124.64 (18)	C20—C15—C16	121.75 (19)
C6—C5—C4	120.53 (17)	C20—C15—O1	119.8 (2)
O4—C5—C4	114.69 (17)	C16—C15—O1	118.42 (19)
C5—C6—C7	119.99 (19)	C15—C16—C17	118.3 (2)
C5—C6—H6	120.0	C15—C16—H16	120.9
C7—C6—H6	120.0	C17—C16—H16	120.9
C2—C7—C6	119.91 (19)	C18—C17—C16	120.6 (2)
C2—C7—H7	120.0	C18—C17—H17	119.7
C6—C7—H7	120.0	C16—C17—H17	119.7
O3—C8—H8A	109.5	C19—C18—C17	120.0 (2)
O3—C8—H8B	109.5	C19—C18—H18	120.0
H8A—C8—H8B	109.5	C17—C18—H18	120.0
O3—C8—H8C	109.5	C18—C19—C20	120.3 (2)
H8A—C8—H8C	109.5	C18—C19—H19	119.9
H8B—C8—H8C	109.5	C20—C19—H19	119.9
C14—C9—C10	121.3 (2)	C15—C20—C19	119.1 (2)
C14—C9—O4	122.70 (19)	C15—C20—H20	120.4
C10—C9—O4	115.7 (2)	C19—C20—H20	120.4
			
C15—O1—C1—O2	1.3 (3)	C5—C6—C7—C2	0.1 (3)
C15—O1—C1—C2	179.29 (18)	C5—O4—C9—C14	50.8 (3)
O2—C1—C2—C7	176.6 (2)	C5—O4—C9—C10	−135.7 (2)
O1—C1—C2—C7	−1.3 (3)	C14—C9—C10—C11	−0.4 (3)
O2—C1—C2—C3	−1.1 (3)	O4—C9—C10—C11	−174.1 (2)
O1—C1—C2—C3	−179.05 (17)	C9—C10—C11—C12	0.7 (4)
C7—C2—C3—C4	−1.1 (3)	C10—C11—C12—C13	−0.4 (4)
C1—C2—C3—C4	176.72 (19)	C11—C12—C13—C14	−0.3 (4)
C8—O3—C4—C3	−5.9 (3)	C10—C9—C14—C13	−0.3 (3)
C8—O3—C4—C5	175.0 (2)	O4—C9—C14—C13	172.91 (19)
C2—C3—C4—O3	−179.20 (19)	C12—C13—C14—C9	0.6 (4)
C2—C3—C4—C5	−0.2 (3)	C1—O1—C15—C20	81.7 (2)
C9—O4—C5—C6	28.7 (3)	C1—O1—C15—C16	−100.9 (2)
C9—O4—C5—C4	−155.67 (19)	C20—C15—C16—C17	−1.6 (3)
O3—C4—C5—C6	−179.50 (19)	O1—C15—C16—C17	−178.9 (2)
C3—C4—C5—C6	1.4 (3)	C15—C16—C17—C18	0.2 (4)
O3—C4—C5—O4	4.6 (3)	C16—C17—C18—C19	0.8 (4)
C3—C4—C5—O4	−174.49 (18)	C17—C18—C19—C20	−0.4 (4)
O4—C5—C6—C7	174.1 (2)	C16—C15—C20—C19	2.0 (3)
C4—C5—C6—C7	−1.3 (3)	O1—C15—C20—C19	179.3 (2)
C3—C2—C7—C6	1.1 (3)	C18—C19—C20—C15	−1.0 (4)
C1—C2—C7—C6	−176.5 (2)		

### Hydrogen-bond geometry (Å, °)

**Table:**

*D*—H···*A*	*D*—H	H···*A*	*D*···*A*	*D*—H···*A*
C8—H8C···O2^i^	0.96	2.52	3.408 (4)	153
C14—H14···O2^ii^	0.93	2.53	3.446 (3)	167
C20—H20···O3^ii^	0.93	2.60	3.468 (3)	155

Symmetry codes: (i) −*x*+1/2, −*y*+3/2, −*z*+1; (ii) −*x*+1, −*y*+1, −*z*+1.
